# Development of a health literacy questionnaire for Taiwanese hemodialysis patients

**DOI:** 10.1186/s12882-016-0266-y

**Published:** 2016-05-31

**Authors:** Chung-liang Shih, Tuan-hsun Chang, Dana A. Jensen, Chiung-hsuan Chiu

**Affiliations:** Ministry of Health and Welfare, 488, Sec. 6, Zhongxiao E. Rd., Nangang Dist., Taipei, Taiwan; National Taiwan University Hospital, 7 Chung Shan S. Rd, Taipei, Taiwan; School of Health Care Administration, Taipei Medical University, 250 Wu-hsing St., Taipei, Taiwan

**Keywords:** Hemodialysis, Health literacy, Health knowledge, Self-care

## Abstract

**Background:**

Dialysis has long been a critical issue in the field of nephrology, though the burden this lifesaving technology places on society can be immense. Effectively increasing the health literacy of hemodialysis patients can be beneficial for their health outcomes and self-care abilities. Thus, the aims of this study are to: (1) develop a health literacy assessment tool in Chinese for patients receiving hemodialysis treatment; (2) assess the health literacy level of the Taiwanese hemodialysis population using the tool developed.

**Methods:**

The questionnaire was developed based on Nutbeam’s three constructs of health literacy and seven sub-constructs identified for the purposes of this study. Experts were consulted for content validity assessment. The questionnaire then was used to conduct a census study at six hospitals and one dialysis clinic that provide hemodialysis treatment in the Taipei area (urban northern Taiwan). To be included, patients must have been at least 18 years old and receiving hemodialysis treatment at the time of the study. 468 eligible respondents were included in the analysis.

**Results:**

The reliability of the tool was 0.81 and the confirmatory factor analysis indicated good construct validity. The mean literacy score for the sample was 19.7 (SD = 4.61) out of a maximum of 26 points, and the median was 21 (range from 6 to 26). Age, education level, primary language, primary caregiver identity, and willingness to receive a transplant were all factors that showed significant association to health literacy level in multiple categories.

**Conclusions:**

The health literacy assessment tool developed in this study is the first health literacy assessment instrument to be made available for a specific patient group in Taiwan. Hemodialysis patients’ knowledge of day-to-day care practices is satisfactory, while their critical literacy is weak.

## Background

Although the literacy rate for the general population in Taiwan is over 98 % [1], this does not indicate that people in Taiwan have adequate *health* literacy. Poor lifestyle is one of the factors associated with the high prevalence of chronic diseases in Taiwan [2–5], and health literacy plays an important role in encouraging positive lifestyle modification. In Taiwan, approximately 370 people per million population require renal dialysis treatment [6]. The total National Health Insurance spending for hemodialysis in 2011 was around 1 billion US dollars, which consumed about 6 % of the entire National Health Insurance budget [7]. However, the health literacy status of these patients was not yet defined.

A statistical report produced by the Taiwanese Department of Health in 2007 ranked renal disease eighth among the top ten causes of death in Taiwan. There were 5099 deaths caused by renal disease and the mortality rate was 22.2 %—a 1.6 % increase from the previous year [1].

The American Medical Association (AMA) has defined health literacy as “the constellation of skills, including the ability to perform basic reading and numerical tasks required to function in the health care environment [8]”. Nutbeam believed that health literacy should include the three typologies of health literacy: basic or functional health literacy, communication or interactive health literacy, and critical health literacy [9]. Health literacy itself is associated with several key factors, such as patient empowerment and successful health self-management [10]. Increased health literacy can minimize the communication gap between health professional and patients. As a result, patients are better able to apply their knowledge when making health-related decisions, minimizing potential problems. Therefore, health literacy is regarded as a necessary skill for bridging the divide between patients and health professionals in a way that adapts to their current health status.

Studies have shown that patients with low health literacy perform unfavorably in a variety of related areas. Several studies indicate that patients with lower reading literacy and oral literacy also tend to be lacking in health-related knowledge [2, 11]. The AMA stated that there is a close relationship between the level of health literacy in the general public and the wasting of medical resources; the extent of medical resource waste for those with lower health literacy is greater than for those with higher health literacy [8]. Results of the National Assessment of Adult Literacy (NAAL) in 1993 showed that approximately 90 million Americans (roughly 36 %) lack the health literacy skills required to obtain appropriate medical information, to assist a diagnosis, or to acquire healthcare services [12]. The NAAL conducted another survey a decade later in 2003; the results showed little progress in the health literacy of Americans. Their knowledge about disease prevention and health maintenance was also inadequate [13]. This study also found that the low health literacy observed led to an additional $7.3 billion in medical expenses [13].

ESRD, hemodialysis, peritoneal dialysis, and kidney transplant patients experienced adjusted mortality rates of 138, 171, 152 and 35 per 1000 patient-years respectively in 2013 [14]. Other studies have observed that the mortality rate of patients who are receiving dialysis is higher than for patients without dialysis treatment [9]. However, only a few health literacy-related studies of patients receiving hemodialysis exist. A study by Ifudu et al. showed that the health literacy and daily self-care of patients receiving hemodialysis were poor in hemodialysis patients in New York and New Jersey [15]. Others have mentioned that the health literacy of patients with late-stage renal disease is often inadequate [16].

Effectively increasing the health literacy of patients can be beneficial for the health and self-care capabilities of patients who are receiving hemodialysis treatment. However, since the current measurement of health literacy is broad and not focused on the investigation of disease-specific literacy, it can be difficult to determine what specific health literacy knowledge patients are lacking. The primary purpose of the study was to develop a health literacy assessment tool in Chinese for patients receiving hemodialysis treatment and to assess the health literacy level of the Taiwanese hemodialysis population using the tool developed.

## Methods

### Developing the tool

**Fig. 1:**
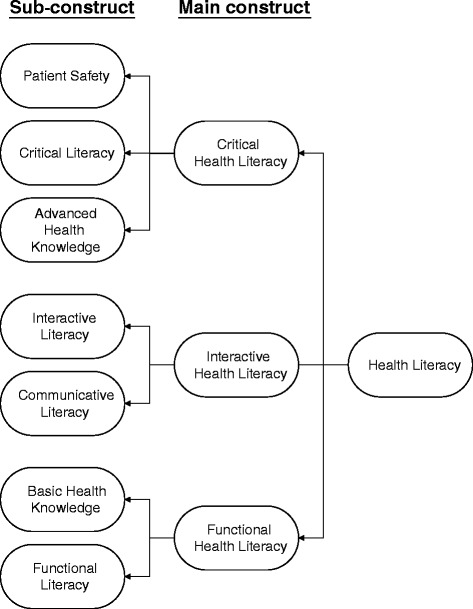
Dimension and sub-dimensions of health literacy in end-stage renal disease

Currently available health literacy tests such as the Test of Functional Health Literacy in Adults (TOFHLA), Wide Range Achievement Test (WRAT), and Rapid Estimate of Adult Literacy in Medicine (REALM) utilize a format that assesses literacy through reading questions and Cloze tests [17–20]. The utility of a health literacy assessment tool is determined by its ability to measure patients’ understanding in the context of their own health, as well as measuring their ability to communicate with their health care provider. The instrument should assess whether patients are able to absorb and comprehend the meaning of health information in the context in which it is encountered. Therefore, based on the relevant literature and knowledge of disease and self-care, we devised an instrument items aligned with the conceptual framework (Fig. 1) and took degree of difficulty into consideration. The total content of readily available patient education information was collected from materials provided by major hospitals in Taiwan, the Bureau of Health Promotion Administration (recently renamed the Health Promotion Administration, Ministry of Health and Welfare) [21], and an expert focus group. The multiple-choice questionnaire was developed based on this information; its validity, relevance, and difficulty level was assessed by nephrologists, nurses, and public health professors.

We constructed a tool divided into two sections: the health literacy assessment and demographic information. There are a total of 52 items in the questionnaire with 26 items in each section. For the health literacy section, this study adapted Nutbeam’s model and incorporated his three key constructs of critical health literacy, interactive health literacy, and functional health literacy. These constructs were further refined to address specific health literacy issues, as shown in Fig. 1. Therefore, the health literacy section includes seven sub-constructs: functional literacy (five items), communicative literacy (four items), interactive literacy (three items), critical literacy (three items), basic health knowledge (four items), advanced health knowledge (five items), and patient safety (two items). One point was awarded for each time respondents selected the correct answer from the four multiple-choice options; no points were awarded if an incorrect response or no response was provided. The maximum total score is 26 and the minimum score is 0. Since foreign caregivers (who are often not fluent in a local language) are very popular in Taiwan, the demographic information section includes questions asking who plays the role of primary caregiver for patients who cannot care for themselves, in order to assess a possible source of poor health literacy. A translated version of the questionnaire items is provided in Appendix 1.

### Validity

After the development of the tool, experts such as nephrologists, nurses, and public health professors were consulted to conduct a content validity assessment and provide constructive comments regarding clarification of descriptions, the appropriateness of questions, and the difficulty level of the questions. All items were reviewed by the experts using a 9-point Likert scale for the validity of each item; a comment section was provided for suggested modifications. Items that were rated below 7 on average were selected for further modification by the team of experts. A pilot test was also conducted with 20 dialysis patients to confirm items’ practicability and items were modified accordingly.

### Setting and study participants

Adult patients over 18 years of age receiving hemodialysis treatment who were able to communicate with interviewers were initially considered for inclusion in this study. Survey data were collected from six hospitals and one hemodialysis clinic in northern Taiwan. The surveys were conducted by interviewers on a one-on-one basis in order to enhance the survey quality and consistency of survey contents. Interviewers received training to improve reliability of the results including discussion of the purpose of this study, detailed explanations and descriptions of methods, and the overall intended contents of the survey session. The six interviewers were able to communicate in both Mandarin Chinese and the Taiwanese dialect in order to facilitate communication with patients using either of these languages. Surveys began in December 2008 at the seven health facilities; we approached a total of 591 patients, and 468 eligible respondents filled out and returned health literacy questionnaires by April 31, 2009. Respondents were excluded if they were unable or unwilling to participate, if they were unable to communicate verbally with the interviewers, or if they chose not to finish the survey process after beginning.

This study was reviewed and approved by the National Taiwan University Hospital (NTUH) IRB, and its approval number was NTUH-200712014R. Interviewers contacted patients at outpatient clinics and obtained respondents’ informed consent through verbal explanation and written in form. Patients’ consent forms were stored in locked cabinets in the PI’s office for 2 years, then shredded and disposed of securely. All data was analyzed on an anonymous basis to ensure respondents’ confidentiality. NTUH’s IRB approved the entire procedure, including the method of obtaining informed consent, data analysis, data storage, and data management.

### Statistical analysis

The surveys were coded and data were analyzed using the statistical software SPSS for Windows 16.0 (SPSS, Chicago, IL, USA) and LISREL 8.7 as analytical tools. The study used descriptive statistics and one-way analysis of variance (ANOVA) to discuss health literacy and explore their connection to individual capabilities and social conditions. Furthermore, construct validity was verified by performing confirmatory factor analysis.

## Results

The mean age of interviewees in the present study was 61.48 years with a standard deviation of 13.74 years, and male respondents accounted for 294 (49.7 %) of the survey respondents. Health literacy was assessed based on multiple-choice questions with four choices but only one correct answer to each question. Incorrect answers, missing answers, and questions with more than one response selected were all scored as incorrect and awarded 0 points, while correct answers were awarded 1 point. In the demographic section of the survey, missing values did not disqualify the full survey case, but these missing values were excluded from analysis. The presence of trained interviewers minimized missing values and incomplete assessments.

### Reliability and construct validity check

In order to check the consistency of responses, split-half methodology was applied to confirm the reliability. Results showed a score of 0.83 overall, which indicated very good consistency. Inter-rater reliability was improved by providing the interviewers’ with consistent training before data collection.

**Fig. 2:**
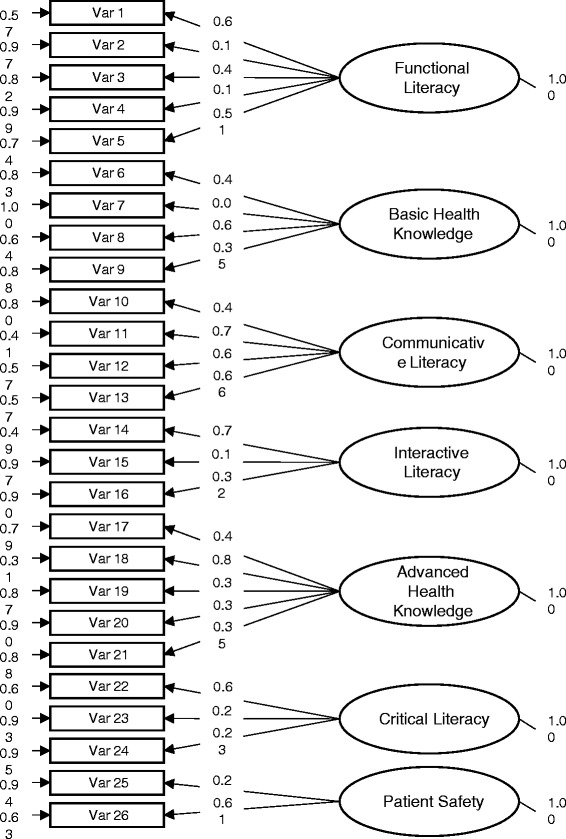
Confirmatory factor analysis on 7-dimensions of health literacy

Confirmatory factor analysis (CFA) was applied to confirm the instrument’s construct validity. Results of goodness of fit showed absolute indices were appropriately high, including GFI (goodness of fit index) = 0.91, AGFI (adjusted goodness of fit index) = 0.9, and CN (critical N) = 285.70. Incremental fit indices were also appropriately high, including NNFI (Non-Normed Fit Index) = 0.93 and CFI (Comparative Fit Index) = 0.94. Parsimony indices were adequate, including PNFI (Parsimony Normed Fit Index) = 0.77 and PGFI (Parsimony goodness of fit index) = 0.72. These indices confirmed high construct validity of this health literacy instrument. CFA indicated very good construct validity for this seven sub-construct health literacy framework as reported in Fig. 2.

### Descriptive statistics

The results showed the average total score was 19.70 (SD = 4.61) out of 26 possible points. The mean score was 3.96 out of 5 (SD = 1.16) for functional literacy, 3.58 out of 4 (SD = 0.76) for basic health knowledge, 3.07 out of 4 (SD = 1.26) for communicative literacy, 2.57 out of 3 (SD = 0.68) for interactive literacy, 3.09 out of 5 (SD = 1.37) for advanced health knowledge, 1.70 out of 3 (SD = 0.74) for critical literacy, and 1.74 out of 2 (SD = 0.47) for patient safety. Among of these 7 sub-constructs, respondents earned relatively high scores in basic health knowledge, interactive literacy, and patient safety but relatively low scores in advanced health knowledge and critical literacy.

### Bivariate analysis

The mean difference in health literacy scores between each group was analyzed for the studied demographic variables, with health literacy as the dependent variable (reported in Table 1).

**Table 1:** Demographic statistics and health literacy of hemodialysis patients in Taiwan in 2009—bivariate analysis by mean and standard deviation. (*n* = 468)

Variable	n	Functional literacy (range: 0–5)	Basic health knowledge (range:0–4)	Communicative literacy (range:0–4)	Interactive literacy (range:0–3)	Advanced health knowledge (range:0–5)	Critical literacy (range:0–3)	Patient safety (range:0–2)	Total score	Median score (IQR)
Gender										
Male	240	3.92 (1.21)	3.58 (0.78)	3.10 (1.22)	2.62 (0.64)	3.10 (1.39)	1.74 (0.77)	1.78 (0.43)	19.83 (4.62)	21 (17, 23)
Female	228	3.99 (1.11)	3.58 (0.74)	3.05 (1.29)	2.51 (0.72)	3.07 (1.35)	1.66 (0.71)	1.69 (0.52)	19.56 (4.62)	21 (16, 23)
*p*-value		*.487*	*.953*	*.683*	*.086*	*.813*	*.248*	*.049*	*.524*	
Age (years)										
< 49	96	4.16 (0.99)	3.75 (0.62)	3.42 (0.89)	2.65 (0.60)	3.67 (1.13)	1.86 (0.69)	1.70 (0.51)	21.20 (3.37)	22 (20, 24)
50–59	138	4.07 (1.10)	3.65 (0.64)	3.20 (1.07)	2.62 (0.64)	3.19 (1.29)	1.80 (0.74)	1.84 (0.39)	20.36 (3.97)	21 (17, 23)
60–69	113	3.89 (1.23)	3.54 (0.83)	3.14 (1.29)	2.58 (0.67)	2.94 (1.38)	1.77 (0.68)	1.70 (0.48)	19.56 (4.96)	21 (16, 23.5)
> 70	114	3.68 (1.25)	3.37 (0.89)	2.56 (1.55)	2.42 (0.79)	2.60 (1.41)	1.39 (0.72)	1.70 (0.51)	17.70 (5.28)	19 (13, 23)
*p*-value		*.011*	*.002*	*<0.001*	*.064*	*<0.001*	*<0.001*	*.034*	*<0.001*	
Education Level										
Illiterate and primary school	184	3.79 (1.27)	3.46 (0.83)	2.72 (1.48)	2.46 (0.75)	2.66 (1.44)	1.49 (0.74)	1.72 (0.48)	18.30 (5.13)	20 (13, 23)
Junior high school	77	4.04 (1.13)	3.60 (0.83)	3.08 (1.27)	2.58 (0.75)	3.19 (1.30)	1.61 (0.65)	1.66 (0.53)	19.77 (4.54)	21 (17, 23)
High school	112	4.04 (1.01)	3.65 (0.72)	3.34 (0.97)	2.66 (0.56)	3.36 (1.21)	1.89 (0.73)	1.76 (0.45)	20.70 (3.95)	22 (19, 24)
College (and above)	94	4.11 (1.10)	3.73 (0.53)	3.45 (0.84)	2.65 (0.58)	3.50 (1.23)	1.96 (0.72)	1.79 (0.72)	21.18 (3.52)	22 (19.75, 24)
* p*-value		*.106*	*.020*	*<0.001*	*.037*	*<0.001*	*<0.001*	*.342*	*<0.001*	
Primary language										
Mandarin	274	3.97 (1.13)	3.62 (0.71)	3.24 (1.17)	2.57 (0.64)	3.26 (1.34)	1.81 (0.72)	1.70 (0.50)	20.17 (4.38)	22 (18, 23)
Taiwanese	192	3.95 (1.21)	3.53 (0.82)	2.84 (1.35)	2.55 (0.73)	2.83 (1.39)	1.57 (0.75)	1.78 (0.44)	19.06 (4.88)	21 (15, 23)
*p*-value		*.860*	*.212*	*.001*	*.744*	*.001*	*.001*	*.085*	*.010*	
Primary caregiver for the patient										
Self or spouse	365	3.98 (1.16)	3.62 (0.72)	3.18 (1.14)	2.62 (0.61)	3.16 (1.34)	1.76 (0.72)	1.76 (0.46)	20.08 (4.28)	21 (17, 23)
Child or a child’s spouse	50	3.82 (1.21)	3.42 (0.86)	2.78 (1.45)	2.38 (0.88)	2.82 (1.45)	1.42 (0.84)	1.60 (0.53)	18.24 (5.29)	20.5 (13, 22.25)
Parents or siblings	15	4.40 (0.83)	3.93 (0.26)	3.27 (0.88)	2.67 (0.72)	3.60 (1.24)	2.00 (0.53)	1.80 (0.41)	21.67 (3.13)	22 (20, 24)
Foreign caregivers or others	39	3.79 (1.22)	3.31 (0.98)	2.38 (1.79)	2.23 (0.84)	2.49 (1.41)	1.46 (0.72)	1.64 (0.54)	17.31 (5.95)	20 (11, 23)
*p-*value		*.291*	*.009*	*.001*	*.001*	*.005*	*.001*	*.071*	*<0.001*	
Willing to receive a kidney transplant										
Yes	166	4.14 (1.09)	3.73 (0.60)	3.40 (0.92)	2.67 (0.59)	3.57 (1.24)	1.89 (0.67)	1.77 (0.43)	21.16 (3.56)	22 (20, 24)
No	259	3.76 (1.21)	3.45 (0.84)	2.83 (1.40)	2.47 (0.73)	2.75 (1.37)	1.58 (0.73)	1.71 (0.50)	18.56 (4.94)	20 (15, 23)
*p*-value		*.001*	*<0.001*	*<0.001*	*.003*	*<0.001*	*<0.001*	*.281*	*<0.001*	

In all cases where significantly varied scores were present, the following trends were observed: males scored higher than females; younger patients scored higher than older patients; more educated patients scored higher than less educated patients; primarily Mandarin-speaking patients scored higher than primarily Taiwanese-speaking patients; patients that were primarily cared for by themselves, a spouse, a parent, or a sibling scored higher than patients that were primarily cared for by a child, a child’s spouse, a foreign caregiver, or others; and patients who were willing to receive a kidney transplant scored higher than patients who were not willing to receive a kidney transplant.

For functional literacy, the variables that showed significant score differences were age and willingness to receive a transplant. For basic health knowledge, the variables that showed significant score differences were age, education level, primary caregiver identity, and willingness to receive a transplant.

For communicative literacy, the variables that showed significant score differences were age, education level, primary language, primary caregiver identity, and willingness to receive a transplant. For interactive literacy, the variables that showed significant score differences were education level, primary caregiver identity, and willingness to receive a transplant.

For advanced health knowledge, the variables that showed significant score differences were age, education level, primary language, primary caregiver identity, and willingness to receive a transplant. For critical literacy, the variables that showed significant score differences were age, education level, primary language, primary caregiver identity, and willingness to receive a transplant. For patient safety, the variables that showed significant score differences were gender and age.

For overall health literacy, the variables that showed significant score differences were age, education level, primary language, primary caregiver identity, and willingness to receive a transplant.

## Discussion

The mean age of the interviewees was 61.48 years (SD = 13.74). According to the Claims Review and Drug Benefit Unit, Bureau of National Health Insurance, the mean age for patients receiving hemodialysis in 2008 was 61.59 years (SD = 13.30) [22]. Therefore, the demographic characteristics of the survey sample are likely representative of the general population of patients receiving hemodialysis in Taiwan.

This study developed a health literacy instrument based on seven sub-constructs for dialysis patients and the construct validity of the instrument was verified by confirmatory factor analysis. Based on the survey results, basic health knowledge received the highest correct answer rate and mean score while critical literacy received the lowest score among the seven sub-constructs of health literacy. Basic health knowledge was associated with knowledge of appropriate diet for patients receiving hemodialysis. While other health literacy instruments assess the ability to spell and pronounce medical terminology, this instrument placed more emphasis on assessing whether patients possessed the necessary dialysis-specific health knowledge to maintain their wellbeing. This means that patients should be more familiar with these issues and able to make the correct choices.

The advanced health knowledge sub-construct covered a variety of formal medical knowledge. Access to this level of medical knowledge was not as readily available as access to basic health knowledge; it required the patient to actively interact with medical staff, as these topics are not commonly encountered in everyday situations. For example, this sub-construct included differentiating between peritoneal dialysis and hemodialysis; peritoneal dialysis is less familiar to (and less commonly used for) dialysis patients in Taiwan, and so the relevant literacy score average was lower.

The critical literacy sub-construct covered the ability to process numerical and analytical data. The critical literacy of patients cared for by their children/child’s spouse or by foreigner caregivers was poorer than others. Since critical literacy is a more abstract form of health literacy, patients should try to obtain the ability to reason and rationalize their findings. Some studies conclude that critical literacy is the ability to think in the abstract and generalize their experience and judgment skills [10]. This survey found that the older a respondents is, the poorer their critical literacy. It could be inferred that this experience is not solely determined by how long the respondents have had their illness, but by how they learn and integrate information based on their experiences. The critical ability of patients who were cared for by their children or a child’s spouse was lowered, and this can be due to the tendency of these patients to rely on their children and the children’s spouses for answers when it comes to the critical literacy sub-construct.

### The significance of health literacy

The bivariate analysis showed that the five demographic variables of age, education level, primary language, primary caregiver identity, and willingness to receive a transplant are associated with the overall health literacy of hemodialysis patients. The association of age and overall health literacy is consistent with the existing literature, which indicates that older patients tend to have poorer health literacy [23]. Furthermore, the findings of better health literacy in more educated patients are also consistent with existing literature.

Curtin and Mapes observed that patients with late-stage renal disease generally have inadequate health literacy [16]. Fraser also found that limited health literacy was common in chronic kidney disease patients through systematic review of several key publications [17, 24]. While it is difficult to make direct comparisons between literacy assessment methods, a 76.6 % overall correct response rate across a variety of relevant areas can be considered a relatively positive sign.

End-stage renal disease is not reversible. To examine these patients and the degree of their health literacy can provide an initial assessment of their knowledge and understanding of their illness as well as their ability to make optimal decision regarding their care. This assessment tool could identify the shortcoming of patients’ health knowledge and health literacy, allowing physicians and nurses to educate them in areas they fall short.

### Limitations

Although this study is the first case of conducting a health literacy assessment specifically targeting Taiwanese hemodialysis patients, the fact that the sampling location was limited to a metropolitan setting may make it difficult to generalize the findings to broader populations. In addition, the present study cannot be directly compared to studies conducted in European and American countries, as the mean score is utilized as the health literacy determination standard in this study; this differs from the partition style (pass, borderline, not pass) used for many assessment tools of health literacy in European and North American studies.

These findings, along with hemodialysis-related health literacy in general, are of particular relevance in populations where hemodialysis or end stage renal disease (ESRD) rates are especially high. Taiwan is an ideal testing ground for hemodialysis-related studies due to the availability of data of the National Health Insurance Research Database (NHIRD) and the fact that Taiwan has the highest global prevalence (2902 per million populations in the year of 2012) of ESRD [25]. Dialysis rates in Taiwan are extremely high, at approximately 1.54 % of the population, compared to 0.13 % in the United States in 2011 [26]. It is well-accepted that poor health literacy leads to additional healthcare costs for a variety of reasons. Since hemodialysis is very costly (both in terms of medical expense and lost productivity) and may continue for years or decades, it is very important to improve the health literacy for this group to minimize further expense. This can benefit the many countries that provide or otherwise financially support hemodialysis services.

One possible complicating factor regarding health literacy in Taiwan is that a high prevalence of the elderly and chronically ill are cared for by foreign caregivers. As of 2003, there are over 195,000 foreigners with temporary residency working as family caregivers in Taiwan, a remarkably high number compared to the overall Taiwanese population of roughly 23 million. Possible language barrier issues can affect communication with patients as well as hospital staff, as many foreign caregivers are not proficient in Chinese. Foreign caregivers may also return to their home country or change which patients they are for, reducing the continuity of care available compared to when patients care for themselves or are cared for by their families. One suggestion for policymakers and dialysis clinic staff is to monitor who is providing care for the patient, and to provide new educational materials and training when presented with a new caregiver. Additionally, dialysis-specific educational materials should be made available in languages frequently spoken by foreign caregivers.

The sampling of this survey focused only on hemodialysis centers in Taipei, the largest city in Taiwan. In contrast to other area of Taiwan, Taipei has high medical accessibility. Hemodialysis patients who live in Taipei have access to more medical and health education resources than patients in other areas. Therefore, in order to assess the health literacy of hemodialysis patients more completely in Taiwan, future studies should deliver this questionnaire to hemodialysis centers and facilities throughout Taiwan.

## Conclusion

The health literacy assessment tool developed in this study is the first health literacy assessment instrument to be made available for a specific patient group in Taiwan, to our knowledge. The concept of this instrument is based on Nutbeam’s health literacy model, and it contains questions based on health literacy topics that hemodialysis patients encounter daily. The health literacy questionnaire has high validity and reliability [9]. Health literacy questionnaires constructed for a particular disease or patient type are necessary to measure their health literacy level as it relates to their specific disease and self-care needs. This specificity is what other general health literacy instruments have been unable to achieve. The research results can help officials tailor their interventions to encourage maximum health literacy and targeted education.

## Appendix 1

### Questionnaire (translated from Chinese)

#### Functional literacy

1. Which of the following statements is INCORRECT for patients who are receiving dialysis treatment?
  A. They should consume a high-protein diet.
  B. It is normal for their stool and urine to appear red.
  C. They should consume plenty of red meat, which is rich in iron.
  D. They should check their skin regularly for lesions or signs of infection.
2. Which of the following is NOT a purpose of dialysis?
  A. To remove excess bodily fluid
  B. To filter out metabolic waste from the blood
  C. To improve the function of the GI system
  D. To balance the body’s electrolytes
3. Which of the following statements regarding artificial blood vessel (Port-A) or arteriovenous fistula care is TRUE?
  A. The patient can touch it or check with a stethoscope—it is functioning normally if it is silent.
  B. The patient’s blood pressure can be measured on the arm with the fistula.
  C. A warm compress should be applied to the site of the fistula to induce blood circulation on the day of dialysis.
  D. Redness at the site is normal and is no cause for concern.
4. Which of the following is a CORRECT way to control blood pressure?
  A. Have a regular lifestyle and eat a low-sodium diet
  B. Stay up late working
  C. Smoking tobacco and drinking
  D. The patient can adjust their dosage of medication depending on their current blood pressure.
5. Which of the following is NOT true for patients with uremia?
  A. They can consume a high protein diet including foods like fish, pork, egg and milk.
  B. They should not be concerned to find red spots in their stool.
  C. They are advised to consume a high-iron diet rich in red meat.
  D. They should regularly check their skin for abrasions and lesions.

#### Basic Health Knowledge

6. Why do you need to control the intake of calcium, potassium and phosphorous?
  A. Excessive intake can cause accumulation of these minerals in the body.
  B. Uremia can decrease the density of bones, so it is important to replenish the body’s stores of calcium.
  C. These minerals are not important for human health.
  D. The body cannot excrete these minerals normally due to decreased renal function.
7. Which type of food should you AVOID when receiving dialysis?
  A. Starfruit
  B. Tofu
  C. Fresh meat such as fish, duck, or chicken
  D. Foods low in phosphorous and potassium
8. Which of the following is the medical reason for monitoring fluid intake?
  A. There is calcium and potassium in water, which should be avoided.
  B. Drinking enough water can help the kidneys get rid of excess waste.
  C. Water can cause edema inside the body due to decreased kidney function and urinary output.
  D. Drinking too much water causes frequent bathroom visits.
9. Which of the following statements regarding fluid consumption is CORRECT for patients receiving dialysis?
  A. Measure out the daily fluid allowance and put it in bottles to be consumed throughout the day.
  B. It is fine to consume water-rich foods such as noodles, curry, and hotpot.
  C. Fruit should be replaced with fresh juice.
  D. The patient should eat more processed and heavily seasoned foods.

#### Communication literacy

10. What should you do when you are uncertain about medical advice or a prescribed treatment regimen?
  A. Doctors are very intelligent and knowledgeable, so it’s normal that I don’t understand and I shouldn’t ask for clarification.
  B. It concerns my health; I have to find out the answer.
  C. I should ask my friends who have had similar problems.
  D. It’s just a minor issue, so I don’t need to bother the doctor about it.
11. What should you do when you are uncertain about your prescribed medication?
  A. I will not take any medication before asking my nephrologist.
  B. I will take it first and monitor my condition.
  C. These medications are normally safe, so it is okay to take them.
  D. I need to take it because my doctor says it is necessary.
12. What should you do with your lab results?
  A. Lab results are too complicated, so I don’t need to understand the results as long as the doctor sees it.
  B. I don’t need to ask about my results because the doctors will tell me themselves.
  C. I will ask about my results even if I don’t understand everything.
  D. Lab results are not important, so I won’t ask about them.
13. What should you do if you feel uncomfortable or unwell after taking a prescribed medication?
  A. I should contact my nephrologist immediately or go to the ER if I can’t reach my nephrologist.
  B. I’m probably just overthinking things and there’s no need to worry.
  C. I should stop taking the medication immediately, but there’s no need to inform the doctor.
  D. I should continue taking the medication because the doctor says I can’t just stop whenever I want.

#### Interactive literacy

14. What should you do when you receive treatment advice from non-medical sources?
  A. Try it immediately
  B. Ask my nephrologist about it
  C. Ask other patients about it
  D. Ask my relatives about it
15. What should you do when you have a GI problem?
  A. Go to the hospital immediately and make sure to tell them that you’re on dialysis
  B. Go to the hospital, but there is no need to mention that you’re on dialysis
  C. Go to the hospital and only describe the GI problem
  D. Go to the pharmacy and buy OTC medication
16. When receiving dialysis treatment, which of the following statements is TRUE?
  A. When I feel better, I can decide to reduce the frequency of dialysis treatment on my own without my doctor’s approval.
  B. I should postpone all medication regimens until dialysis treatment finishes.
  C. I should discuss any medications I’m taking with my nephrologist.
  D. Feeling nauseated after dialysis is a normal symptom, and I do not need to tell the doctor.

#### Advanced health knowledge

17. Which of the following is NOT a common complication in dialysis patients?
  A. High blood pressure
  B. Cardiovascular disease
  C. Stroke
  D. Kidney stones
18. Which of the following therapies can treat End-Stage Renal Disease (ESRD)?
  A. Traditional Chinese medicine and acupuncture
  B. Kidney transplantation
  C. Cocktail therapy
  D. Medication
19. Which of the following statements about dialysis is FALSE?
  A. Hemodialysis uses an artificial filter machine (artificial kidney) to filter excess fluid and waste products from the blood stream.
  B. Peritoneal dialysis uses the patient's peritoneum (located in the abdomen) as a membrane across which excess fluids and waste products are exchanged from the blood.
  C. Patients receiving hemodialysis need to visit a dialysis center 2-3 times a week to receive treatment.
  D. Hemodialysis is more effective and has fewer complications than peritoneal dialysis.
20. Which of the following statements about kidney transplant is CORRECT?
  A. I can only receive a kidney from blood relatives.
  B. As long as someone is willing to donate their kidney, I don’t need to wait and can proceed with transplantation immediately.
  C. The condition of the donor and recipient must match in order for a transplantation to proceed.
  D. Once the donor is willing to donate, there is no checkup needed.
21. Which of the following is an APPROPRIATE precaution when taking oral
  A. Before a dialysis session, I should stop taking all hypertensive medications.
  B. When I feel better, I can stop taking the oral medication.
  C. I don’t need to take my medication on a regular schedule. I only need to take it when I have elevated blood pressure.
  D. Dialysis will remove the medication from my system, so I need to take oral medications again after the dialysis.

#### Critical literacy

22. If your dry weight is 50 kg, which of the following is NORMAL weight gain?
  A. 0-1 kg
  B. 2-3 kg
  C. 4-5 kg
  D. 5-6 kg
23. If the doctor tells you that you can gain 2 kg after two dialysis treatments, your urine output is about 200cc, and you experience natural water loss of about 500cc, what is your ideal amount of water intake per day?
  A. 700cc
  B. 1700cc
  C. 2700cc
  D. 3700cc
24. Which of the following statements about self-care is CORRECT for patients receiving dialysis?
  A. Patients should have a regular exercise schedule of at least 30 minutes 3 times a week.
  B. I can consume traditional Chinese herbs to help with my disease.
  C. I should use low-sodium salt when cooking.
  D. Drinking a cup of wine can help with sleeping.

#### Patient safety literacy

25. Which of the following statements regarding fall prevention is TRUE?
  A. After a dialysis treatment, even if I am feeling dizzy and nauseated I can get off the bed without assistance.
  B. I should gradually get off of the bed or change positions, and I can stand up only after I am able to sit up without experiencing dizziness.
  C. Feeling dizzy is a normal and does not require extra precautions.
  D. To prevent foot injuries, I can wear shoes that are larger than my regular size.
26. Which of the following statements about infection prevention in dialysis patients is CORRECT?
  A. Before dialysis, I can wash the site with soapy water or clean water.
  B. After installation of an artificial blood vessel, I should keep the wound clean.
  C. I should only wash my body with a wet cloth to avoid excess water on the wound.
  D. When there is redness or signs of swelling around the wound, I should go to the hospital immediately. If there is bleeding, I should not apply pressure to the wound.

## Abbreviations

AGFI: See Adjusted Goodness Of Fit Index
AMA: See American Medical Association
CFA: See Confirmatory Factor Analysis
CFI: See Comparative Fit Index
CN: See Critical
ESRD: See End-stage renal disease
GFI: See Goodness of Fit Index
NAAL: See National Assessment of Adult Literacy
NHIRD: See National Health Insurance Research Database
NNFI: See Non-Normed Fit Index
NTUH: See National Taiwan University Hospital
One-way ANOVA: See One-way Analysis Of Variance
REALM: See Rapid Estimate of Adult Literacy in Medicine
SD: See Standard Deviation
PGFI: See Parsimony Goodness Of Fit Index
PNFI: See Parsimony Normed Fit Index
TOFHLA: See Test of Functional Health Literacy in Adults
WRAT: See Wide Range Achievement Test
